# TRENDS IN RECEIPT OF PAID FAMILY CARE AMONG OLDER ADULTS WITH FUNCTIONAL IMPAIRMENT IN THE UNITED STATES, 2011–2022

**DOI:** 10.1093/geroni/igae098.1692

**Published:** 2024-12-31

**Authors:** Jennifer Reckrey, Yifan Liu, Yiqing Qian, Katherine Ornstein

**Affiliations:** Icahn School of Medicine at Mount Sinai, Icahn School of Medicine at Mount Sinai, New York, United States; Johns Hopkins University, Baltimore, Maryland, United States; Johns Hopkins University, Baltimore, Maryland, United States; Johns Hopkins University, Baltimore, Maryland, United States

## Abstract

While caregiving is frequently conceptualized as either paid care (e.g., home health aides, home care workers) or family care (e.g., spouses, children), family members may also receive payment to provide care (i.e., paid family care) through state-level Medicaid-waiver programs or through private arrangements within families. This study examines trends in paid family care prevalence over time and identifies factors associated with receipt of paid family care among older adults with functional impairment using the National Health and Aging Trends Study. Prevalence of paid family care increased slightly over time, with 7.0%, 5.9%, and 8.1% of the sample receiving paid family caregiving in 2011, 2015, and 2022 respectively. In 2022, 27.8% of those with any form of paid help received paid family care; 27.1% was funded privately and 72.9% was funded by other payers like Medicaid. As compared to those with a non-family paid helper, older adults with paid family care were more likely to be female (78.1% vs. 55.6%, p=0.028), Black/non-Hispanic (29.2% vs. 11.5%, p<0.001), and receive Medicaid (62.5% vs. 33.1%, p<0.001). They reported more chronic conditions (4.1 vs 3.2, p=0.012), impairment in more self-care tasks (3.18 vs 2.44, p=0.043), and more total weekly hours of care (20.0 vs 11.2, p<0.001). Paid family care makes a meaningful contribution to total care, particularly among those with high care needs. Future work examining paid family care both within and outside of the Medicaid system should explore the impact of paid family care on older adults and their larger family unit.

